# Quality control of protein import into mammalian mitochondria

**DOI:** 10.1002/pro.70585

**Published:** 2026-04-28

**Authors:** Madeleine Goldstein, Laurie Lee‐Glover, Hilla Weidberg

**Affiliations:** ^1^ Life Sciences Institute, Department of Cellular and Physiological Sciences University of British Columbia Vancouver British Columbia Canada

**Keywords:** mitochondrial dysfunction, mitochondrial protein import, Proteostasis, quality control mechanisms, stress responses

## Abstract

Mitochondrial function depends on the continuous import of hundreds of nuclear‐encoded proteins. Targeting and translocation of mitochondrial proteins is a multistep process that is inherently vulnerable to defects in cytosolic quality control systems as well as perturbations in mitochondrial protein import machinery and organelle function. Failure of mitochondrial protein import has dual consequences: it compromises mitochondrial biogenesis and activity, and it poses a cytosolic proteotoxic threat due to the accumulation of unimported precursor proteins. Accordingly, mitochondrial protein import defects are detrimental to cellular homeostasis and are associated with a wide range of disorders, including metabolic and neurodegenerative diseases. Cells therefore rely on layered quality control systems that monitor mitochondrial protein biogenesis and mitigate stress arising from mislocalized mitochondrial proteins. In this review, we summarize recent progress in understanding pathways that modulate mitochondrial protein import and the fate of unimported proteins in mammals. We highlight cytosolic and mitochondrial protein quality control mechanisms and discuss how import defects are translated into cellular stress responses and mitochondrial protective programs to restore cellular and mitochondrial homeostasis.

## 1 INTRODUCTION

Mitochondria are the site for various metabolic pathways, including oxidative phosphorylation (OXPHOS), which produces ATP (Suomalainen & Nunnari, 2024). The hundreds of proteins that comprise the mitochondrial proteome are indispensable for organelle function and homeostasis (Busch et al., 2023; Pfanner et al., 2025). While mitochondria are self‐sufficient in transcription and protein translation, mitochondrial DNA (mtDNA) encodes only 13 proteins in humans. Consequently, continuous import of proteins is required to supply the remainder of the mitochondrial proteome, which is encoded by nuclear DNA (Busch et al., 2023; Morgenstern et al., 2021; Pfanner et al., 2025).

Proteins enter the mitochondria through the translocase of the outer membrane (TOM) complex and are subsequently delivered to their appropriate compartments via various import machineries, such as the translocase of the inner membrane 23 (TIM23) (for broader overviews of mitochondrial protein import, see Busch et al., 2023; Endo & Wiedemann, 2025; Pfanner et al., 2025). Most mitochondrial proteins contain internal or N‐terminal mitochondrial targeting signals (MTSs). These signals are imperative for protein targeting and translocation and are recognized by the import receptors TOM20, TOM22, and TOM70 at the mitochondrial surface (Busch et al., 2023; Pfanner et al., 2025). The import rate of mitochondrial proteins is thought to depend on their specific MTS “strength”, suggesting that these signals contribute to the regulation of protein biogenesis (Rödl et al., 2025; Schäfer et al., 2022; Yan et al., 2026).

Upstream mechanisms that target nascent mitochondrial precursors to mitochondria remain largely unknown. Some proteins were suggested to translocate into the organelle co‐translationally via mitochondria‐associated ribosomes (Chang et al., 2025; Gold et al., 2017; Kellems et al., 1975; Kellems & Butow, 1974; Lesnik et al., 2014; Luo et al., 2025; Marc et al., 2002; Sylvestre et al., 2003; Williams et al., 2014; Zhu et al., 2025). However, the majority of proteins are thought to be post‐translationally targeted to mitochondria (Bykov et al., 2020). Together with their co‐chaperones, cytosolic members of the general chaperone families, Heat Shock Protein 90 and 70 (Hsp90 and Hsp70) play a role in this process (Fan et al., 2006; Juszkiewicz et al., 2025; Komiya et al., 1996; Young et al., 2003). Yet additional factors and the molecular details by which precursors are maintained in a translocation‐competent state are poorly characterized.

Mitochondrial protein import is impaired in various metabolic and neurodegenerative disorders, such as diseases caused by mutations in import machinery components (Palmer et al., 2021; Pfanner et al., 2025). Moreover, diverse forms of mitochondrial dysfunction inhibit protein import, including damage to mtDNA, respiratory chain defects, or any perturbation that disrupts the inner membrane potential, leading to membrane depolarization (Busch et al., 2023; Fu et al., 2023; Kim et al., 2023; Palmer et al., 2021; Peng et al., 2015). Similarly, imbalanced mitochondrial proteostasis can impair protein import by saturating chaperones that normally associate with TIM23 to drive protein translocation into the matrix and promote folding (Lee‐Glover & Shutt, 2024; Michaelis et al., 2022; Nargund et al., 2012; Sutandy et al., 2023). As protein import efficiency is tightly coupled to mitochondrial function, it can serve as a sensitive indicator of organelle health.

Beyond its impact on mitochondria, inefficient protein import poses a proteotoxic threat to cells due to the accumulation of unimported precursor proteins outside the organelle. These precursors often contain exposed hydrophobic regions that promote aggregation and overload cytosolic quality control and degradation pathways (Wright et al., 2001; Peng et al., 2015; Cenini et al., 2016; Liu et al., 2018; Boos et al., 2019; Liu et al., 2019; Nowicka et al., 2021; Schlagowski et al., 2021; Wang et al., 2021; Kim et al., 2023; Amponsah et al., 2025; reviewed in: Maruszczak et al., 2023; Balzarini et al., 2025; Pfanner et al., 2025). Cytosolic accumulation of unimported precursors was shown to trigger mitochondrial stress responses that aim to restore cellular and mitochondrial homeostasis (Boos et al., 2019; Kim et al., 2023; Krämer et al., 2023; Lee‐Glover & Shutt, 2024; Sutandy et al., 2023; Wang & Chen, 2015; Weidberg & Amon, 2018; Wrobel et al., 2015; Wu, Meyer, et al., 2019a).

Collectively, these and other studies over the past decade have transformed our understanding of mitochondrial protein import. This has led to a shift from a largely linear view of protein targeting and translocation to a more integrated perspective in which protein import is tightly coupled to cellular proteostasis and stress signaling networks. Rather than functioning as an isolated process, mitochondrial protein import is now recognized as both a regulator and a sensor of cellular homeostasis, with defects in import triggering adaptive responses that coordinate cytosolic and mitochondrial quality control pathways. Many of these conceptual advances were first uncovered in model organisms such as budding yeast, where genetic and biochemical approaches revealed extensive crosstalk between protein translocation, degradation, and stress response pathways.

These findings have laid the foundation for our current understanding of mitochondrial protein quality control in mammalian systems, where our knowledge is rapidly expanding. In this review, we highlight recent insights into mitochondrial protein quality control pathways in mammals. We focus on mechanisms that maintain mitochondrial protein biogenesis and adapt organelle functions, as well as signaling pathways that sense and repair cellular damage arising from perturbations in mitochondrial protein import. When not mentioned otherwise, the findings summarized here were discovered using cultured mammalian cells; studies using human tissues or mice are explicitly indicated.

## 2 QUALITY CONTROL OF MITOCHONDRIAL PRECURSORS IN THE CYTOSOL

### 2.1 Cytosolic surveillance of mitochondrial protein import by chaperones

**FIGURE 1:**
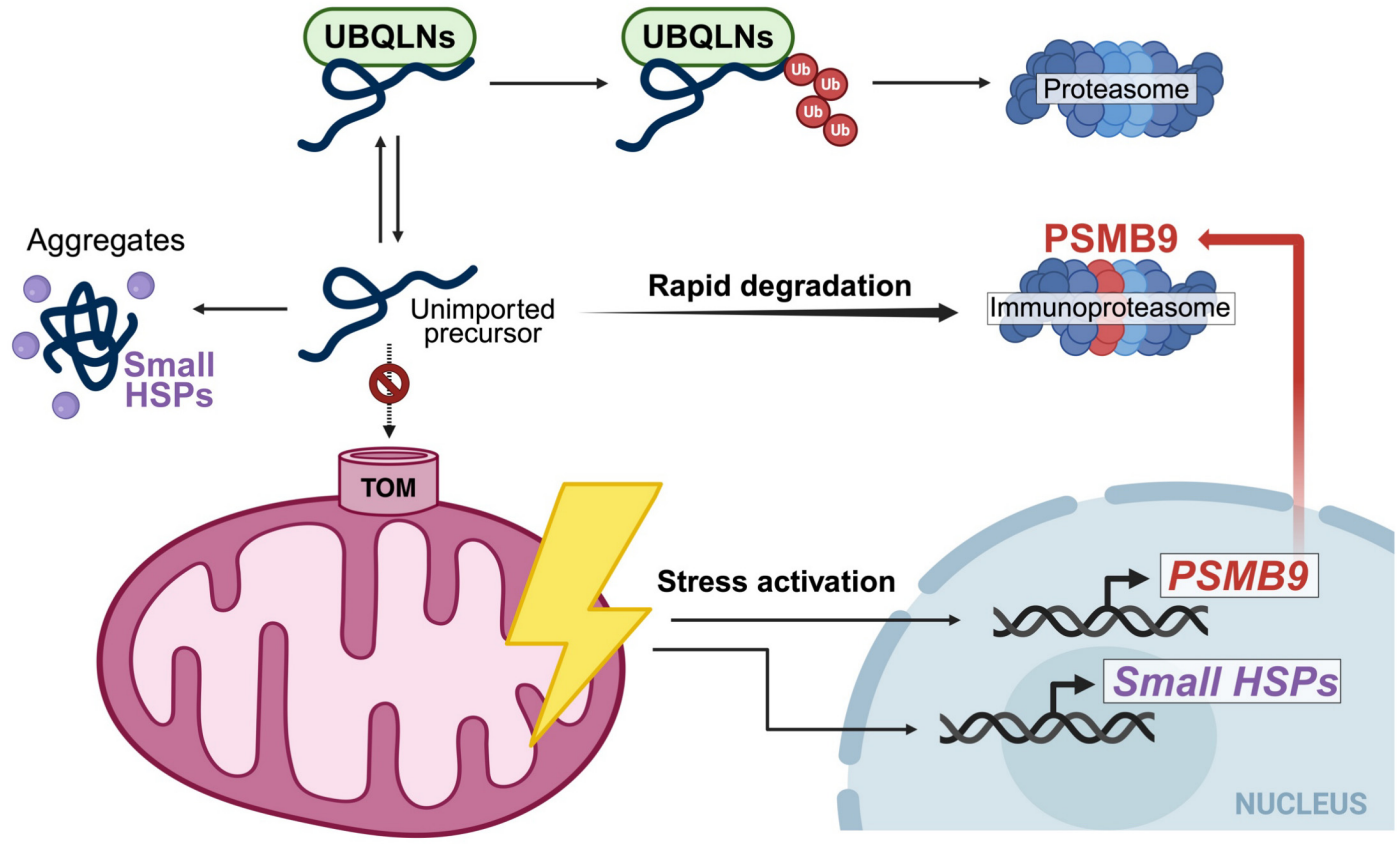
Cytosolic management of unimported mitochondrial precursors. When mitochondrial protein import is impaired, precursors can accumulate in the cytosol where they can aggregate along with small Heat Shock Protein (HSP). Transmembrane domains of mitochondrial membrane proteins associate with ubiquilins (UBQLNs), which can promote their proteasomal degradation upon prolonged binding. Mitochondrial stress induces the expression of small HSPs to improve chaperone activity in the cytosol, as well as the immunoproteasome subunit, PSMB9. PSMB9‐containing proteasomes enhance the cellular degradation capacity and facilitate the rapid elimination of unimported mitochondrial precursors.

When imported efficiently, mitochondrial proteins are exposed to the cytosol only for a limited time. HSP70, HSP90, and additional chaperones are known to interact with these precursors at early stages of biogenesis, prior to import (Fan et al., 2006; Juszkiewicz et al., 2025; Komiya et al., 1996; Young et al., 2003). Under mitochondrial stress that reduces protein import efficiency, prolonged association of these chaperones can maintain precursors in a translocation‐competent state and prevent their aggregation (Juszkiewicz et al., 2025). Recent work highlights a role for small HSPs, including HSPB1 and HSPH1, in ameliorating the proteotoxicity of unimported mitochondrial proteins (Figure 1) (Kim et al., 2023; Wang et al., 2021). Small HSPs bind substrates through exposed hydrophobic domains, and members of this family co‐aggregate with mitochondrial unimported proteins in yeast (Krämer et al., 2023; Kuzu et al., 2025). Similarly, HSPB1 and HSPH1 associate with protein aggregates in mammalian models with impaired mitochondrial protein import, including cells lacking complex I subunits (Kim et al., 2023). In these models, aggregates are also enriched in MTS‐containing mitochondrial precursors (Kim et al., 2023). Protein aggregation is enhanced in the absence of HSPB1, suggesting that small HSPs maintain mistargeted mitochondrial proteins in a soluble, non‐toxic state (Kim et al., 2023). Expression of HSPB1 and HSPH1 is induced when mitochondrial protein import is compromised, consistent with their protective role (Dabir et al., 2013; Kim et al., 2023). Similar upregulation of HSPs was observed in mouse skeletal muscles upon moderate overexpression of the mitochondrial carrier protein Ant1 (Wang et al., 2021). Excess carrier protein levels exceed the capacity of the mitochondrial import machinery, and their hydrophobic properties render them particularly prone to aggregation (Liu et al., 2019; Wang et al., 2021). Overall, the function of small HSPs may provide a temporal buffer that allows cells either to restore mitochondrial import capacity or to redirect unimported proteins toward degradation pathways (Haidar et al., 2019; Zhang et al., 2010). Under certain stresses, such as intra‐mitochondrial protein aggregation, small HSPs can translocate to the mitochondrial intermembrane space (IMS) where they facilitate protein folding, thereby also protecting against mitochondrial proteotoxicity (Adriaenssens et al., 2023; Fan et al., 2022). The localization of HSPs to the IMS was validated in various mammalian cell lines as well as in primary human‐derived lymphoblasts, human‐derived fibroblasts, mouse retinas, and mouse heart tissue (Adriaenssens et al., 2023).

Mitochondrial dysfunction is not the sole cause of protein import defects. Proteotoxic conditions in mammals, including accumulation of neurodegeneration‐associated proteins, inhibit mitochondrial protein import (Cenini et al., 2016; Di Maio et al., 2016; Li et al., 2010; Needs et al., 2023; Schlagowski et al., 2021; Sergeant et al., 2003; Trushina et al., 2004; Yano et al., 2014). Impaired protein import has been detected in mitochondria isolated from brain tissues of various rat and mouse disease models, as well as in human brain and spinal cord tissues from patients with Parkinson's, Huntington's, and amyotrophic lateral sclerosis (Di Maio et al., 2016; Li et al., 2010; Needs et al., 2023; Yano et al., 2014). The accumulation of disease‐associated proteins can also promote aggregation of newly synthesized proteins en route to mitochondria, as demonstrated in multiple systems, including human brain tissue from Alzheimer's and Huntington's disease patients (Cenini et al., 2016; Schlagowski et al., 2021; Sergeant et al., 2003; Trushina et al., 2004). This aggregation of mitochondrial proteins depletes the organelle of essential factors and causes mitochondrial damage, further exacerbating protein import defects (Amponsah et al., 2025; Hussain et al., 2021; Kim et al., 2023; Liu et al., 2018; Liu et al., 2019; Liu et al., 2023; Nowicka et al., 2021; Ruan et al., 2017; Wang et al., 2021; Wrobel et al., 2015). Proteotoxic stress is also a hallmark of aneuploid cells, due to imbalanced protein complex stoichiometry that overwhelms cellular protein quality control systems (Amponsah et al., 2025). Indeed, MTS‐containing proteins were identified in aggregates isolated from aneuploid cells (Amponsah et al., 2025). Intriguingly, these aggregates also contain the autophagic receptor p62 but are not subjected to autophagic degradation, suggesting that they primarily function to sequester harmful proteins and limit proteotoxicity (Amponsah et al., 2025).

The fate of aggregated mitochondrial proteins in human cells remains largely unknown. While some aggregated proteins may be cleared by proteasomal and possibly autophagic degradation, others may persist for extended periods. In yeast, aggregation of MTS‐containing proteins into structures termed MitoStores was shown to be reversible, allowing renewed attempts at mitochondrial import once the damage is resolved (Krämer et al., 2023). Further studies are needed to determine whether a similar mechanism exists in mammalian cells.

### 2.2 Role for the ubiquitin‐proteasome pathway in eliminating unimported mitochondrial precursors

Protein degradation constitutes a crucial mechanism for elimination of unimported mitochondrial proteins and for protection against proteotoxicity (Boos et al., 2019; Bragoszewski et al., 2013; Finger et al., 2020; Haakonsen et al., 2024; Itakura et al., 2016; Kim et al., 2023; Kowalski et al., 2018; Liu et al., 2019; Mohanraj et al., 2019; Schäfer et al., 2022; Schulte et al., 2023; Wang & Chen, 2015; Whiteley et al., 2017; Wright et al., 2001; Wrobel et al., 2015). Ubiquilins (UBQLNs) chaperone mitochondrial membrane proteins in the cytosol by binding their transmembrane domains (Itakura et al., 2016). Dynamic association of UBQLNs with these substrates enables their mitochondrial import upon release. However, prolonged binding promotes recruitment of E3 ubiquitin ligases, leading to substrate ubiquitination and subsequent proteasomal degradation (Itakura et al., 2016). When UBQLN function is impaired, unimported mitochondrial proteins accumulate in the cytosol, leading to proteotoxic stress and protein aggregation (Itakura et al., 2016). Overall, UBQLNs surveil mitochondrial proteostasis by maintaining the balance between mitochondrial protein import and degradation under basal conditions and, presumably, also when protein import is impaired (Figure 1).

A specialized form of the proteasome, the immunoproteasome, is induced by inflammatory cytokines and incorporates the catalytic subunit PSMB9 in place of the constitutive β1 subunit (Zou et al., 2025). Expression of PSMB9 is upregulated in models of mitochondrial stress with defective protein import, leading to enhanced proteasome activity (Kim et al., 2023; Kodroń et al., 2025). This system enables rapid clearance of irreparably unimported mitochondrial proteins, thereby limiting excessive aggregation and proteotoxicity (Figure 1) (Kim et al., 2023; Kodroń et al., 2025). Interestingly, the immunoproteasome assembles approximately four times faster than the constitutive proteasome, making it well suited for acute stress adaptation (Heink et al., 2005). Additional studies are needed to determine which unimported mitochondrial precursors are immunoproteasome substrates and whether ubiquilins cooperate with immunoproteasomes to promote efficient degradation during mitochondrial stress. Together, these systems likely cooperate to ensure that mislocalized mitochondrial proteins are efficiently buffered, sequestered, and cleared. Notably, proteasomal degradation can also be deleterious under certain conditions, as demonstrated in models of mitochondrial diseases caused by pathogenic OXPHOS subunit mutants (Kodroń et al., 2025; Mohanraj et al., 2019). In this context, proteasome inhibition increases mitochondrial abundance of mutant proteins and improves mitochondrial function, suggesting that proteasome modulation may represent a potential therapeutic strategy for certain mitochondrial diseases (Kodroń et al., 2025; Mohanraj et al., 2019).

### 2.3 Ribosomal quality control of nascent mitochondrial precursors

Unlike most quality control factors that sense the folding state of proteins, the ribosomal quality control (RQC) pathway senses the state of translation as a readout for protein fidelity (Joazeiro, 2019). This pathway targets nascent polypeptides for proteasomal degradation, thereby preventing the release of aberrant and potentially toxic proteins from ribosomes (Joazeiro, 2019). Various perturbations that impair translation, including defective ribosomes and aberrant mRNAs, can lead to ribosome stalling and initiate the RQC pathway (Joazeiro, 2019). In yeast, a mitochondrial RQC (mitoRQC) was shown to protect mitochondrial function by eliminating aberrant nascent polypeptides that are translated at the mitochondrial surface (Izawa et al., 2017). Inhibition of this pathway results in the addition of C‐terminal extensions to faulty proteins prior to their import, leading to their aggregation inside mitochondria (Izawa et al., 2017). C‐terminal extensions of mitochondrial proteins are similarly toxic in other organisms. In mammals, mitochondrial membrane depolarization impairs translation termination of precursors such as the complex I subunit NDUFS3 by disrupting the termination factor eRF1 and the ribosome recycling factor ABCE1 (Wu et al., 2018; Wu, Tantray, et al., 2019b). This defect promotes the generation of NDUFS3 C‐terminal extensions, resulting in either cytosolic aggregation or, upon import, interference with complex I function (Wu, Tantray, et al., 2019b). While mammalian mitoRQC is not yet fully characterized, recent studies have identified components involved in this pathway in multiple systems including mice and human primary fibroblasts (Geng et al., 2024; Lavie et al., 2023). These studies demonstrated that the E3 ubiquitin ligases ZNF598 and FBXL6 mediate degradation of stalled translation products (Geng et al., 2024; Lavie et al., 2023). Notably, FBXL6 substrates include defective subunits of mitochondrial ribosomes (Lavie et al., 2023), which, if imported, could impact ribosome assembly and compromise mitochondrial translation.

## 3 CONTROL OF PROTEIN IMPORT AT THE MITOCHONDRIAL SURFACE

### 3.1 Surveillance of protein import at the TOM complex

USP30 and March5 are outer mitochondrial membrane (OMM) proteins that constitutively monitor mitochondrial protein import at the TOM complex (Phu et al., 2020). March5 is an E3 ubiquitin ligase that ubiquitinates mitochondrial precursors to structurally block their translocation and promote their proteasomal degradation (Phu et al., 2020). USP30 deubiquitinases these precursors, counteracting March5 activity and restoring protein import (Ordureau et al., 2020; Phu et al., 2020). March5 and USP30 were also shown to modulate import efficiency by directly targeting TOM subunits for degradation in both cell models and mice (Phu et al., 2020). The balance between March5 and USP30 activity is therefore necessary to maintain appropriate protein import. Loss of USP30 disrupts this balance, leading to the accumulation of ubiquitinated import intermediates that may create a bottleneck at the mitochondrial entry site (Phu et al., 2020).

**FIGURE 2:**
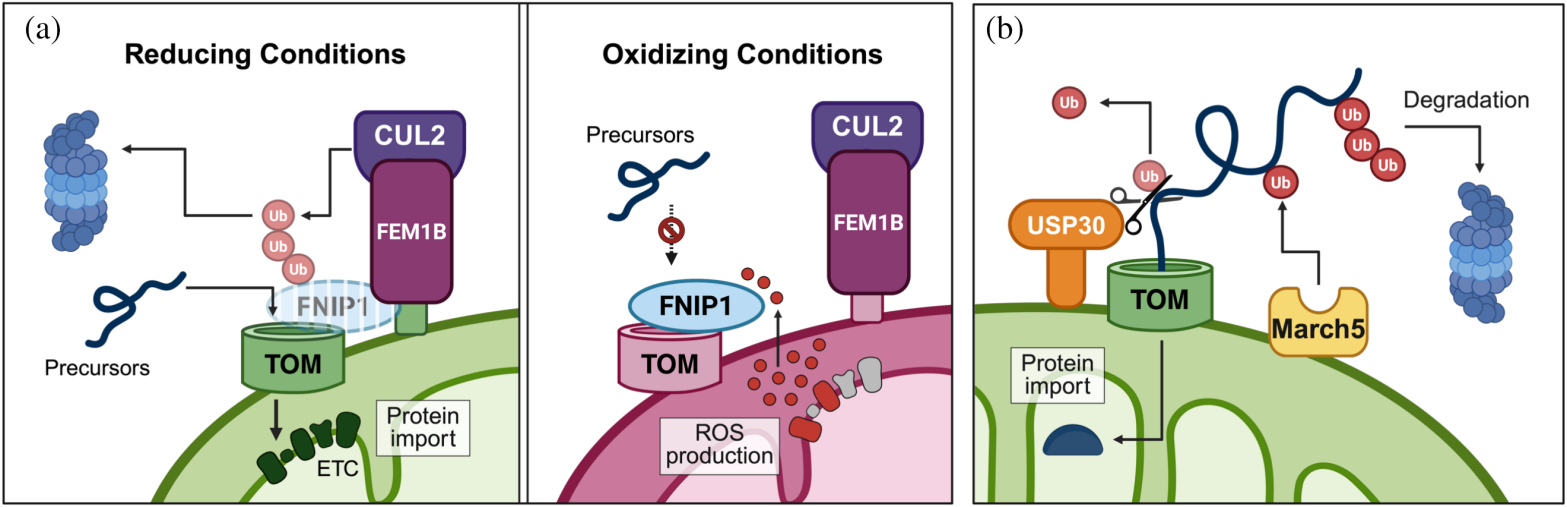
Tuning of protein import on the mitochondrial surface. (a) FNIP1 modulates protein import by binding to the TOM22 import receptor to prevent biogenesis of electron transport chain (ETC) complexes and excessive ROS production (right panel). When ROS levels decline, reduced FNIP1 is recognized by the ubiquitin ligase complex CUL2FEM1B and degraded (left panel). Removal of FNIP1 enhances mitochondrial protein import. (b) The balance between March5 and USP30 activity monitors protein import at TOM. March5 ubiquitinates mitochondrial precursors to prevent their translocation and promote proteasomal degradation. USP30 counteracts March5 by removing ubiquitin chains, thus allowing import to proceed.

Although the regulation of March5 activity remains largely unknown, modulation of protein import by E3 ubiquitin ligases may provide a mechanism for adapting mitochondrial metabolism. Such a mechanism was suggested for the Cullin 2–RING ligase complex with the FEM1B adaptor (CUL2^FEM1B^) and its substrate folliculin‐interacting protein 1 (FNIP1) (Manford et al., 2020; McMinimy et al., 2024). Both CUL2^FEM1B^ and FNIP1 associate with the cytosolic surface of the OMM through binding to TOM20 and TOM22, respectively. FNIP1 interaction with TOM22 fine‐tunes the import of matrix and IMS proteins, likely by limiting their accessibility to TOM22. By monitoring protein import, FNIP1 restricts the biogenesis and activity of electron transfer chain complexes, thereby preventing excessive reactive oxygen species (ROS) production (Manford et al., 2020; Manford et al., 2021; McMinimy et al., 2024). When ROS levels decline, cysteine residues within FNIP1 become reduced, enabling CUL2^FEM1B^ binding and subsequent FNIP1 degradation (Manford et al., 2020; Manford et al., 2021). Removal of FNIP1 from TOM enhances protein import and mitochondrial function (McMinimy et al., 2024). Mitochondrial CUL2^FEM1B^ also regulates turnover of the fusion factor PLD6, linking this E3 ligase to mitochondrial dynamics in addition to redox sensing (Raiff et al., 2026). Together, the March5 and CUL2^FEM1B^ ubiquitination systems act as gatekeepers, adjusting protein import rates to fine‐tune mitochondrial activity and cellular homeostasis (Figure 2).

**FIGURE 3:**
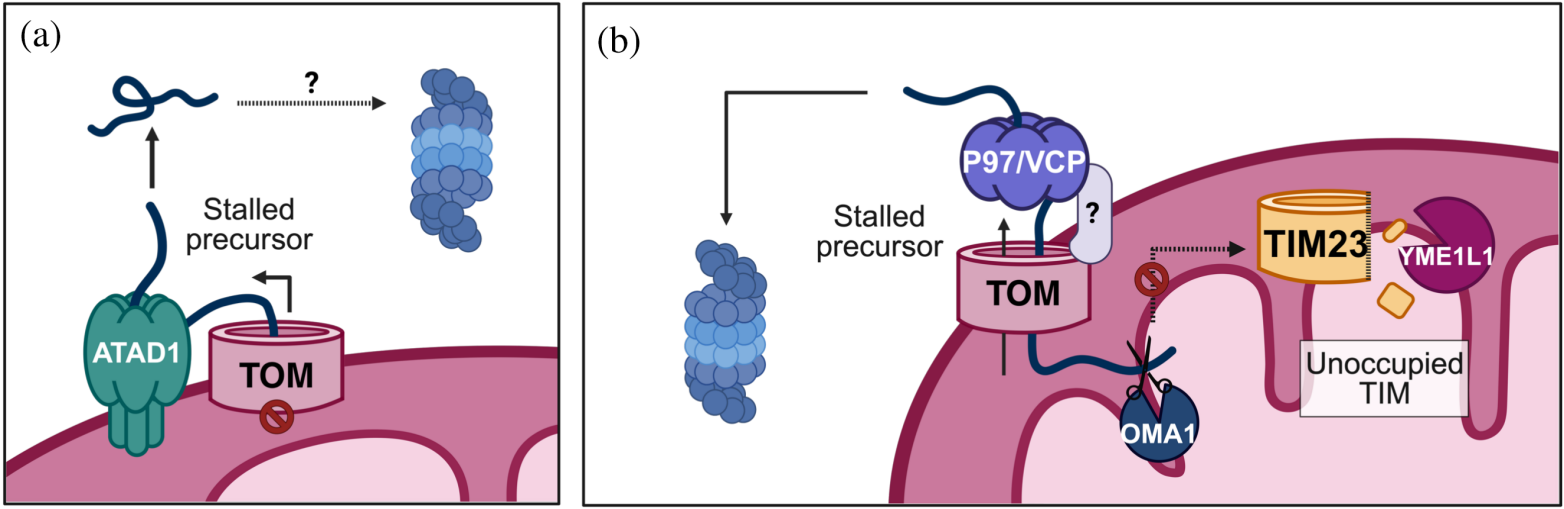
Clearance of stalled mitochondrial precursor intermediates. (a) During mitochondrial protein import stress, the ATAD1 ATPase localizes to clogged TOM to facilitate the removal of stalled precursors, likely enabling their degradation by the proteasome. (b) Stalled precursor removal from clogged TOM is also facilitated by the p97/VCP ATPase and an unknown adaptor before handoff to the proteasome for degradation. Cloggers that stall in both TOM and the inner membrane activate OMA1, thus severing them from the IMM and allowing extraction by p97/VCP. Clogging of TOM can leave the inner membrane translocase, TIM23, unoccupied, leading to YME1L1‐mediated degradation of TIM23 subunits.

ATAD1 (ATPase family AAA domain‐containing protein 1) is an ATPase that maintains mitochondrial protein import fidelity in both yeast and mammals (Kim et al., 2024; Weidberg & Amon, 2018). This function is particularly important when protein import is impaired and mitochondrial precursors become trapped within TOM (Kim et al., 2024). Both native and mutant mitochondrial proteins can stall while translocating through TOM, including pathogenic mutants of the carrier ANT1, as demonstrated in cellular systems and mice (Coyne et al., 2023; Kim et al., 2024). Localized to the OMM with its ATPase domain facing the cytosol, ATAD1 extracts stalled proteins and facilitates their degradation (Basch et al., 2020; Kim et al., 2024; Wang & Walter, 2020; Weidberg & Amon, 2018). By clearing clogged translocases, ATAD1 enables subsequent rounds of protein import (Figure 3a; Kim et al., 2024). In addition, ATAD1 safeguards OMM integrity by removing mistargeted proteins, including tail‐anchored and peroxisomal proteins (Chen et al., 2014; Fresenius & Wohlever, 2019; Nuebel et al., 2021; Wang & Walter, 2020). While not directly linked to protein import, this role of ATAD1 in OMM quality control likely supports proper function of protein import components associated with this membrane.

In yeast, the mitochondrial protein translocation‐associated degradation (mitoTAD) pathway also mediates clearance of proteins stalled in TOM (Mårtensson et al., 2019). In this pathway, the conserved AAA‐ATPase Cdc48 extracts stalled precursors and targets them for proteasomal degradation (Mårtensson et al., 2019). MitoTAD depends on the adaptor protein Ubx2 that is best known for its role in ER‐associated degradation (ERAD) but also localizes to the OMM (Mårtensson et al., 2019). FAF2/UBXD8, the human ortholog of Ubx2, exhibits a similar dual localization. The mitochondrial pool of FAF2/UBXD8 associates with the TOM complex and March5 and regulates cell death and mitochondrial quality control (Zheng et al., 2022). This function is mediated through recruitment of the mammalian Cdc48 ortholog p97/VCP, promoting degradation of pro‐apoptotic and mitophagy‐related factors (Zheng et al., 2022). Notably, p97/VCP was recently demonstrated to remove blocked “cloggers” from TOM (Krakowczyk et al., 2024). However, the identity of the adaptor that recruits p97/VCP to clogged TOM is yet to be identified.

### 3.2 Clearance of outer membrane import components

Mitochondria‐derived vesicles (MDVs) play an important role in maintaining mitochondrial homeostasis (König & McBride, 2024). MDVs remove unwanted and damaged components such as oxidized proteins and can contain both outer and inner mitochondrial membranes (König et al., 2021; Soubannier et al., 2012; Todkar et al., 2021; Vasam et al., 2021). Once formed, MDVs can be targeted to lysosomes, where their contents are degraded. MDV formation can be triggered in a PTEN‐induced kinase 1 (PINK1) and Parkin‐dependent mechanism (McLelland et al., 2014). Since activation of PINK1 is known to occur upon its failed import, a link between protein import defects and MDV formation is plausible (Jin et al., 2010; Matsuda et al., 2010; Narendra et al., 2010). It is an attractive possibility that MDV‐mediated removal of import machinery components regulates mitochondrial protein import capacity and clears damaged translocases. Indeed, entire TOM complexes have been identifies in these vesicles (König et al., 2021; König & McBride, 2024).

A different membrane remodeling process involves the shedding of large OMM‐derived structures, termed SPOTs (structures positive for OMM), which were identified in cells infected with the human parasite *Toxoplasma gondii* (Li et al., 2022). SPOT formation depends on parasite association with TOM70 on host mitochondria, which perturbs TOM70‐dependent import (Li et al., 2022). SPOT formation was also observed upon overexpression of an OMM‐targeted GFP, suggesting that this process may represent a general mechanism to preserve OMM integrity (Li et al., 2022). SPOTs may have evolved to regulate mitochondrial protein import broadly or to selectively modulate specific pathways, such as TOM70‐dependent import, thereby influencing carrier protein abundance and mitochondrial metabolic functions.

Rather than selectively removing components from damaged mitochondria, entire organelles can instead be eliminated either intracellularly by mitophagy or transferred intercellularly to neighboring cells (Narendra et al., 2010; Needs et al., 2024; Yang et al., 2023). Cells with defective mitochondrial protein import have been shown to transfer mitochondria via tunneling nanotubes in exchange for healthy mitochondria (Needs et al., 2024). Similarly, a quality control process called mitocytosis expels damaged mitochondria from the cell periphery in cellular models and mice (Jiao et al., 2021). Previous work reported higher mitochondrial activity in perinuclear mitochondria compared to those near the cell periphery, as evidenced by increased membrane potential and TOM density (Wurm et al., 2011). These observations raise the possibility that mitochondria with reduced import capacity and function are preferentially expelled by mitocytosis. What determines the intracellular localization of damaged mitochondria, and under which circumstances it is beneficial to expel these organelles rather than target them for degradation, is unclear. Furthermore, how cells distinguish mitochondria that are sufficiently healthy to warrant repair via unclogging mechanisms or MDVs, and whether such repair machineries are less active at the cell periphery, are important questions for future investigation.

## 4 QUALITY CONTROL OF IMS AND INNER MEMBRANE PROTEIN IMPORT

### 4.1 Regulation of the TIM23 complex

While mechanisms that regulate protein import and quality control at the mitochondrial surface are well studied, our understanding of how protein import is monitored and regulated at the inner mitochondrial membrane (IMM) remains limited. Recent studies suggest that targeted degradation of import machinery components may act as a key regulatory mechanism for protein translocation across the IMM (Hsu et al., 2025; MacVicar et al., 2019; Rainbolt et al., 2013). Such mechanisms have not been identified in yeast, suggesting that higher eukaryotes may possess additional regulatory layers downstream of the TOM complex. In mammals, the core TIM23 complex, which mediates protein translocation across the IMM, consists of the TIM23 subunit and either TIM17B (one of two isoforms) or its stress‐sensitive homolog, TIM17A (Jain et al., 2025; Sinha et al., 2014). Under various stress conditions, both TIM23 and TIM17A subunits are degraded by the IMM protease YME1L, leading to attenuation of mitochondrial protein import (Hsu et al., 2025; MacVicar et al., 2019; Rainbolt et al., 2013). YME1L‐mediated degradation can be triggered by a synthetic TOM “clogger”, which contains an N‐terminal IMM transmembrane domain and a tightly folded C‐terminus exposed to the cytosol (Hsu et al., 2025). This “clogger” is laterally inserted from the TIM23 translocase into the IMM and thus stalls within TOM but does not remain in TIM. Hsu et al. suggested that under such conditions, YME1L activity is induced, possibly by sensing unoccupied TIM23 translocases. Elimination of the TIM17A and TIM23 subunits may prevent further damage by inhibiting additional insertion of “cloggers” into the IMM, as this step requires the TIM23 translocase (Figure 3b).

Conversely, factors such as OCIAD1 increase TIM17A stability by protecting it from YME1L‐mediated degradation (Elancheliyan et al., 2024). OCIAD1 can interact with the prohibitin complex, which promotes the assembly of both TIM17A‐ and TIM17B‐containing TIM23 complexes (Elancheliyan et al., 2024). Maintaining a balance between degradation and biogenesis of TIM23 complexes is likely essential to preserve a basal level of import capacity, which is crucial for recovery after stress resolution. This requirement for balance may also extend to the relative abundance of TIM17A‐ versus TIM17B‐containing TIM23 complexes, which may facilitate adaptation to specific metabolic and stress conditions. Indeed, the selective degradation of TIM17A during stress suggests that the identity of the TIM17 subunit may influence specific functional properties of the translocase.

In addition, mitochondrial proteases can resolve TOM blockage by cleaving clogged precursors. Expression of a similar IMM‐embedded “clogger” has been shown to disrupt membrane polarization and activate the inner membrane metalloprotease OMA1 (Krakowczyk et al., 2024). Activated OMA1 cleaves the “clogger”, severing it from the IMM and enabling its extraction from TOM by p97/VCP (Figure 3b; Krakowczyk et al., 2024). Protein import through the TIM23 complex is also regulated by degradation of the import motor DNAJC15 (Kroczek et al., 2026; Sinha et al., 2014). This OMA1‐mediated degradation selectively decreases the import of OXPHOS components (Kroczek et al., 2026) and may potentially serve as a mechanism to adjust mitochondrial metabolism in response to stress. While these recent discoveries have begun to shed light on how protein import across the IMM is monitored, several key aspects of this process remain poorly understood. It is unclear how occupancy and stalling of the translocase are sensed, what signals trigger the selective degradation of the translocase subunits, and how the balance between different TIM23 complex pools is maintained. As major gaps in our understanding of TIM23 subunit function and organization persist, a deeper understanding of this complex and how it facilitates protein translocation will likely aid in addressing these questions.

### 4.2 Regulation of protein import into the IMS

**FIGURE 4:**
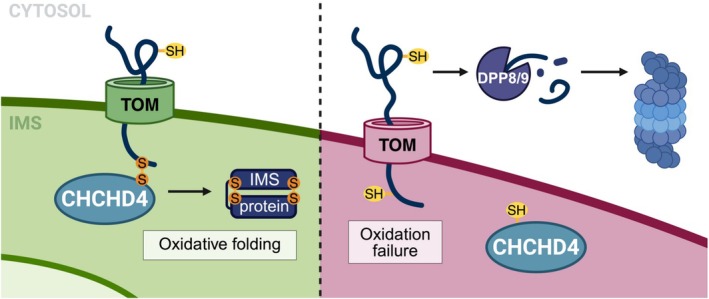
Regulation of IMS protein import via the disulfide relay. IMS‐targeted proteins rely on CHCHD4‐mediated oxidation for their proper folding and retention in the IMS. Upon import through TOM, CHCHD4 forms intermediate disulfide bonds with the IMS substrate before facilitating the formation of its intramolecular disulfide bonds. Failed oxidative folding results in retro‐translocation of the IMS substrate through TOM into the cytosol, followed by processing by the DPP8/9 proteases and proteasomal degradation. DPP8/9 can also compete with the import of IMS precursors in the cytosol to regulate IMS protein import.

CHCHD4 (also known as MIA40) is a central component of the disulfide relay machinery that mediates the import of small IMS proteins (Busch et al., 2023). CHCHD4 functions as both a receptor and an oxidoreductase in the IMS, importing proteins that contain characteristic cysteine motifs (e.g., CX3C or CX9C) and form two disulfide bonds upon maturation. During import, CHCHD4 forms an intermediate intermolecular disulfide bond with its substrates (Busch et al., 2023; Habich et al., 2019). As translocation proceeds, disulfide bonds are formed intramolecularly within the substrate, effectively trapping the mature folded protein in the IMS (Busch et al., 2023). Failure of oxidation prevents proper folding and leads to retrotranslocation of these IMS proteins through TOM, followed by their proteasomal degradation in the cytosol (Bragoszewski et al., 2015; Habich et al., 2019). The capacity of the disulfide relay pathway is a rate‐limiting step in IMS protein import, as evidenced by the increased abundance of mitochondrial IMS proteins in CHCHD4‐overexpressing cells (Schlagowski et al., 2021). Thus, CHCHD4 serves as a redox quality control factor that regulates import and prevents the accumulation of mutated or damaged proteins that are unable to fold in the IMS (Figure 4).

Substrates of the disulfide relay pathway extend beyond canonical IMS proteins. A two‐step import process has been described for the complex I assembly factor NDUFAF8, which acquires disulfide bonds in the IMS before reaching the matrix (Peker et al., 2023). Excess NDUFAF8 exceeds the oxidative capacity of CHCHD4 and is therefore degraded either in the IMS by YME1L or in the matrix by ClpP protease (Peker et al., 2023). This unique mechanism couples mitochondrial oxidative folding with complex I biogenesis, suggesting dynamic regulation of IMS import in response to respiratory demand. Similarly, APE1, a component of the mitochondrial base excision repair pathway, is imported via a related route. Following its import into the IMS by CHCHD4, APE1 translocates into the matrix via TIM23 at an increased rate during oxidative stress (Barchiesi et al., 2020).

IMS protein import is also regulated in the cytosol by the ubiquitin‐proteasome pathway (Finger et al., 2020; Radke et al., 2008). IMS proteins, including substrates of the disulfide relay pathway, are targets of the cytosolic serine proteases dipeptidyl peptidases 8 and 9 (DPP8/9) (Finger et al., 2020). DPP8/9 facilitates the N‐terminal cleavage of these proteins, targeting them for efficient degradation via the N‐end‐rule pathway (Figure 4; Finger et al., 2020). This process can regulate mitochondrial biogenesis and function by preventing import into the IMS and can also promote degradation of retrotranslocated proteins to prevent their re‐entry into the mitochondria. It has also been proposed to block unwanted cytosolic activity of mistargeted IMS proteins.

In the cytosol, IMS proteins can overload cytosolic quality control pathways, especially under proteotoxic stress conditions such as the presence of polyglutamine (polyQ) proteins (Schlagowski et al., 2021). Notably, enhancing IMS protein import through increased CHCHD4 expression suppresses polyQ aggregation and toxicity (Schlagowski et al., 2021). Thus, coordinated regulation between mitochondrial import and cytosolic quality control pathways is required for IMS protein homeostasis, and the balance between these processes can determine cellular proteostasis.

## 5 SENSORS OF MITOCHONDRIAL PROTEIN IMPORT DEFECTS

As mitochondrial protein import is highly susceptible to mitochondrial damage, defects in import can serve as an early warning signal of mitochondrial dysfunction (Lee‐Glover & Shutt, 2024; Pfanner et al., 2025). Consistent with this idea, mitochondrial stress responses in diverse organisms have been shown to be triggered by proteins that act as stress messengers when their entry into mitochondria is blocked (Abudu et al., 2019; Aras et al., 2020; Fessler et al., 2020; Fiorese et al., 2016; Guo et al., 2020; Killackey et al., 2022; Narendra et al., 2010; Shpilka & Haynes, 2018; Yuan et al., 2026).

### 5.1 Activation of the UPR^mt^ by unimported mitochondrial proteins

**FIGURE 5:**
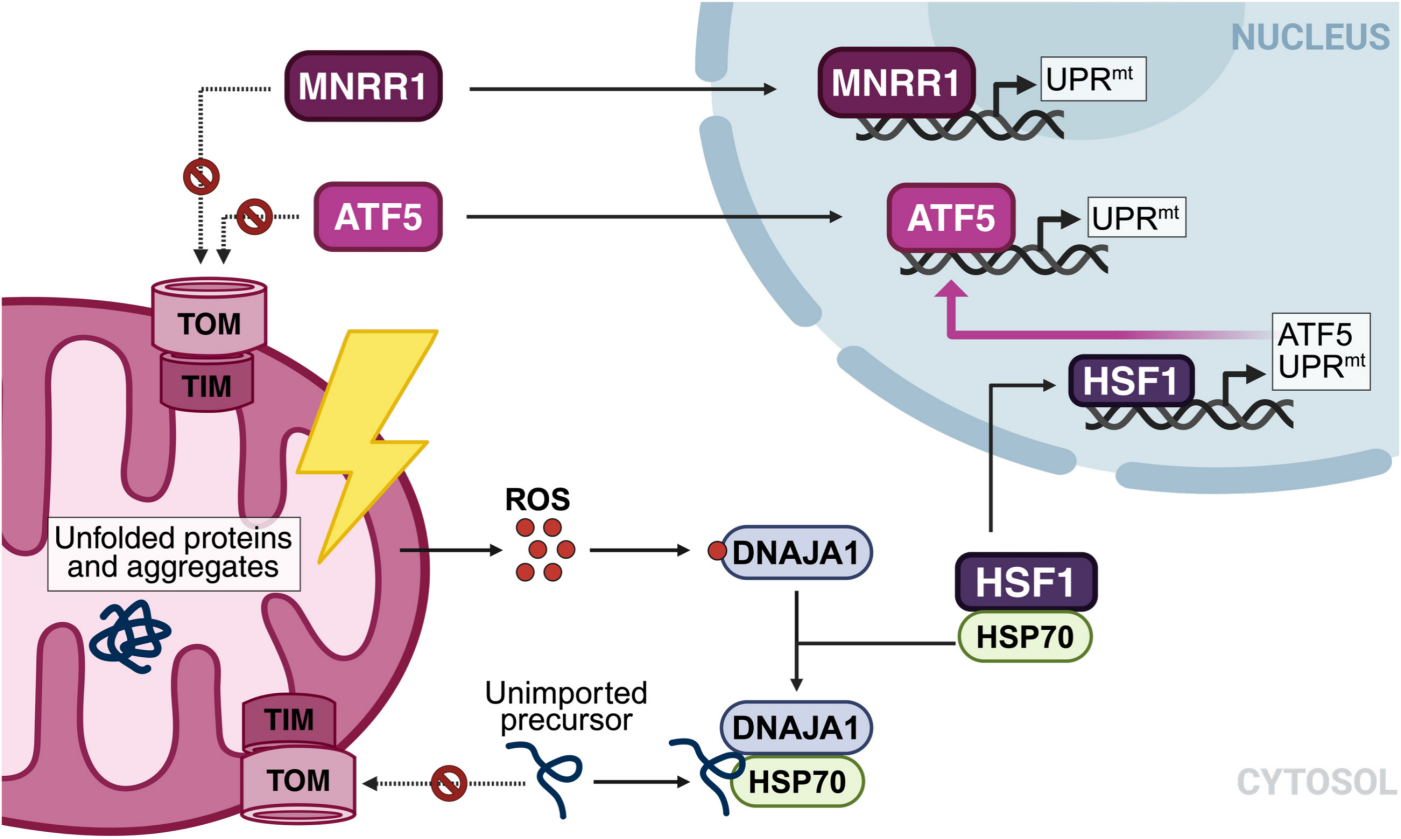
Induction of the mitochondrial Unfolded Protein Response (UPRmt). Mitochondrial damage, including proteotoxic accumulation of unfolded proteins, impairs the import of ATF5 and MNRR1. Under these conditions, ATF5 and MNRR1 translocate to the nucleus to activate the expression of UPRmt genes, thereby increasing mitochondrial capacity to import and fold proteins. Similar defects also activate HSF1; ROS accumulation oxidizes the cytosolic co‐chaperone DNAJA1 to enhance its affinity for HSP70. DNAJA1 and HSP70 are then recruited to unimported mitochondrial precursors. This titrates HSP70 away from HSF1, thus allowing HSF1 to enter the nucleus and activate the transcription of UPRmt genes as well as ATF5, which further amplifies the UPRmt.

Quality control of proteins within mitochondria is regulated by organelle‐specific chaperones and proteases (Baker et al., 2025). This process is crucial, as the accumulation of unfolded proteins and aggregates inside mitochondria can disrupt organelle function and impair protein import (Baker et al., 2025). Such proteostasis perturbations trigger the mitochondrial unfolded protein response (UPR^mt^), a transcriptional program that upregulates mitochondrial genes to restore the organelle's capacity to import and fold proteins (Aldridge et al., 2007; Shpilka & Haynes, 2018; Zhao, 2002). In *Caenorhabditis elegans*, the transcription factor ATFS‐1 regulates the UPR^mt^ (Shpilka & Haynes, 2018). ATFS‐1 contains both an MTS and a nuclear localization signal and is normally imported into the mitochondrial matrix (Nargund et al., 2012; Shpilka et al., 2021). Defects that compromise mitochondrial import of ATFS‐1 result in its redirection to the nucleus and induction of the UPR^mt^ (Nargund et al., 2012; Shpilka et al., 2021; Shpilka & Haynes, 2018). Although the activation mechanisms of the UPR^mt^ are less well characterized in mammals, several transcription factors were suggested to regulate this response, including Activating Transcription Factor 5 (ATF5), Mitochondrial Nuclear Retrograde Regulator 1 (MNRR1, also known as CHCHD2), and Heat Shock Factor 1 (HSF1) (Aldridge et al., 2007; Aras et al., 2020; Fiorese et al., 2016; Katiyar et al., 2020; Sutandy et al., 2023; Zhao, 2002). Notably, ATF5 is positively regulated by the integrated stress response (ISR), which is also activated by mitochondrial damage, suggesting crosstalk between cellular stress pathways (Zhou et al., 2008). Similar to ATFS‐1, both MNRR1 and ATF5 were shown to translocate to the nucleus and activate the UPR^mt^ upon failure of their mitochondrial import (Figure 5; Fiorese et al., 2016; Aras et al., 2020).

Recent work characterizing an HSF1‐mediated UPR^mt^ pathway provides an additional link between mitochondrial protein import defects and UPR^mt^ activation (Katiyar et al., 2020; Sutandy et al., 2023). HSF1 activation in the cytosol requires integration of two signals: accumulation of unimported mitochondrial precursors and mitochondrial release of ROS. ROS initiate this process by oxidizing the cytosolic J‐domain co‐chaperone DNAJA1, thereby enhancing its affinity for HSP70. DNAJA1 and HSP70 are subsequently recruited to exposed hydrophobic regions of unimported mitochondrial precursors (Juszkiewicz et al., 2025; Sutandy et al., 2023). As unimported precursors accumulate, cytosolic HSP70 becomes increasingly occupied by these substrates, reducing its availability to bind and inhibit HSF1 (Sutandy et al., 2023). Upon release, HSF1 translocates into the nucleus, where it induces expression of UPR^mt^ target genes (Figure 5; Zhao, 2002; Aldridge et al., 2007; Katiyar et al., 2020; Sutandy et al., 2023). This regulation is consistent with the previously observed chaperone titration mechanism, in which HSF1 activation is repressed by chaperones (Zheng et al., 2016). Interestingly, under mitochondrial stress, HSF1 also induces expression of ATF5, suggesting that the DNAJA1/HSF1 pathway acts upstream of ATF5 (Sutandy et al., 2023). Altogether, mammalian UPR^mt^ regulators seem to function as stress‐responsive switches that link mitochondrial protein import efficiency to protective transcriptional programs. These responses promote restoration of mitochondrial function and protein import, including import of the regulators themselves, which could lead to UPR^mt^ termination once homeostasis is re‐established.

### 5.2 Defective DELE1 import and activation of the ISR

**FIGURE 6:**
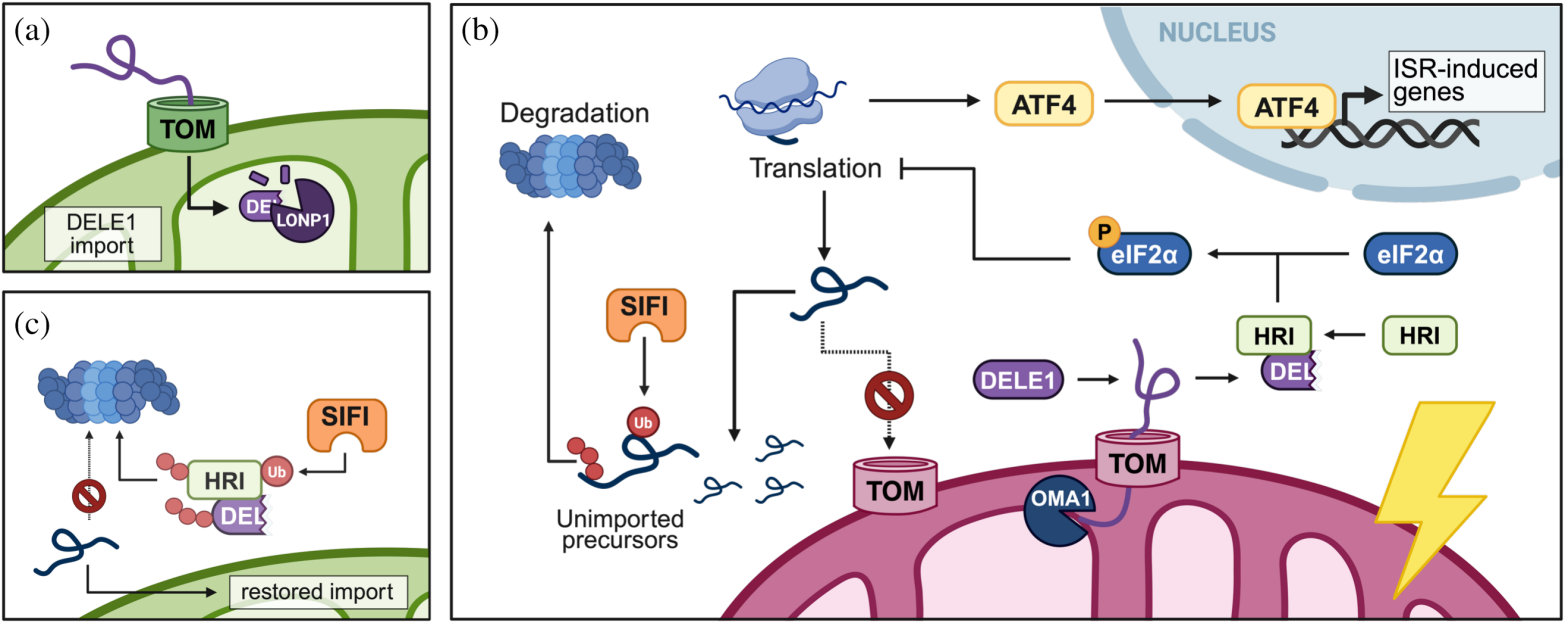
Mechanism of Integrated Stress Response (ISR) induction and silencing. (a) Under nonstress conditions, DAP3 Binding Cell Death Enhancer 1 (DELE1) is constantly imported into the mitochondrial matrix where it is degraded by the LONP1 protease. (b) Upon protein import stress, DELE1 translocation is impaired, resulting in its cleavage by the inner membrane protease OMA1. Cleaved DELE1 is released into the cytosol and binds to the HRI kinase, thus activating it. Activated HRI phosphorylates the eukaryotic initiation factor eIF2α, leading to reduced global translation and selective synthesis of stress‐responsive proteins, including the transcription factor ATF4. ATF4 stimulates the expression of ISR‐induced genes which regulate cell survival, amino acid metabolism, and redox homeostasis. In parallel, the stress causes mitochondrial precursors to accumulate outside the organelle, where they are subjected to degradation by the SIFI ubiquitin ligase and the proteasome. (c) Once mitochondrial protein import is restored, SIFI is free to act upon DELE1 and HRI to mediate their degradation and terminate the ISR.

DELE1 (DAP3 Binding Cell Death Enhancer 1) is a key mediator linking mitochondrial stress to activation of the ISR, a conserved cellular program that enables adaptation to various stress conditions (Fessler et al., 2020; Guo et al., 2020; Lin et al., 2024). Under normal conditions, DELE1 is targeted to the mitochondrial matrix, where it is continuously degraded by the protease LONP1 (Sekine et al., 2023). However, during mitochondrial dysfunctions that inhibit protein import, such as membrane depolarization, mtDNA defects, or iron depletion, DELE1 mitochondrial translocation is impaired (Fessler et al., 2020; Fessler et al., 2022; Fu et al., 2023; Guo et al., 2020; Sekine et al., 2023). Under these conditions, DELE1 is cleaved by OMA1 and subsequently released into the cytosol, where it activates the heme‐regulated inhibitor kinase (HRI) (Fessler et al., 2020; Guo et al., 2020; Sekine et al., 2023; Yang et al., 2023). Other forms of DELE1 were shown to activate HRI, including the full‐length DELE1 and a smaller fragment, arising from HtrA2‐mediated processing in the cytosol (Bi et al., 2024; Fessler et al., 2022). Once activated, HRI phosphorylates the eukaryotic initiation factor 2 alpha (eIF2α), resulting in global attenuation of protein synthesis (Yang et al., 2023). This reduces the burden on the mitochondrial protein import machinery and helps restore protein import (Yang et al., 2024). At the same time, the ISR selectively enhances the translation of stress‐responsive mRNAs, including ATF4 (Yang et al., 2023). ATF4 is a transcription factor that induces genes involved in amino acid metabolism, redox homeostasis, and, under conditions of severe or prolonged stress, apoptosis (Quirós et al., 2017; Yang et al., 2023). Thus, DELE1 acts as a sensor of mitochondrial protein import defects that relays signals to the cytosolic translation machinery to activate an adaptive cellular response (Figure 6).

Mitochondrial protein import efficiency also governs termination of DELE1‐HRI signaling. Shutoff of this pathway is mediated by the Silencing Factor of the ISR (SIFI) ligase complex, which ubiquitinates DELE1 and HRI, targeting them for proteasomal degradation (Haakonsen et al., 2024; Yang et al., 2025). Through this mechanism, SIFI limits the duration and magnitude of ISR signaling, enabling cells to return to baseline once the damage is resolved. However, as long as the mitochondrial stress persists and unimported mitochondrial precursors are retained in the cytosol, DELE1 and HRI are relatively protected. In this context, unimported precursors compete with DELE1 and HRI for SIFI binding, thereby sustaining ISR signaling (Figure 6; Haakonsen et al., 2024). Importantly, SIFI is not activated during ER‐induced ISR, highlighting its specificity for the DELE1‐HRI axis and suggesting that unique termination mechanisms operate across different ISR branches (Haakonsen et al., 2024).

Additional layers of regulation may control DELE1 processing within mitochondria. Such a role has been proposed for CHCHD2 and CHCHD10, which form heterodimers in the IMS and whose mutations are associated with a spectrum of neurodegenerative diseases (Anderson et al., 2019; Liu et al., 2020; Ruan et al., 2022; Shammas et al., 2022). Activation of OMA1, cleavage of DELE1, and induction of the ISR have all been observed in mice expressing pathogenic CHCHD10 mutations, as well as upon combined CHCHD2/CHCHD10 loss in cells and mouse models (Anderson et al., 2019; Liu et al., 2020; Ruan et al., 2022; Shammas et al., 2022). Notably, CHCHD2 and CHCHD10 were shown to bind OMA1 and are proposed to inhibit its activity, suggesting a direct role of these proteins in regulating ISR induction and other OMA1‐dependent processes (Ruan et al., 2022). However, the mechanisms governing CHCHD2/CHCHD10 interaction with OMA and how they are influenced by mitochondrial state remain largely unknown.

Although the ISR can promote recovery from mitochondrial stress and restore cellular homeostasis, sustained activation of this pathway can be deleterious. Indeed, chronic ISR signaling has been implicated in neurodegenerative disorders and aging‐related pathologies (Bravo‐Jimenez et al., 2025). Thus, mechanisms that suppress DELE1–HRI signaling are likely essential to prevent prolonged ISR activation, even in the presence of persistent mitochondrial damage.

### 5.3 Defective PINK1 import and activation of mitophagy

When protective responses are insufficient to restore mitochondrial function and protein import, dysfunctional mitochondria are eliminated through selective autophagic turnover, or mitophagy. PINK1 acts as a sensor of mitochondrial dysfunction to designate damaged mitochondria for degradation (Jin et al., 2010; Matsuda et al., 2010; Narendra et al., 2010). Similar to DELE1, PINK1 monitors protein import. Under basal conditions, PINK1 is imported to the IMM and cleaved by the mitochondrial protease presenilin‐associated rhomboid‐like protein (PARL) (Jin et al., 2010). Cleaved PINK1 is released into the cytosol, where it is cleared by the proteasome (Yamano & Youle, 2013). However, during mitochondrial stress, PINK1 import across the TIM23 complex is impaired, leading to its accumulation on the OMM and subsequent recruitment of Parkin to initiate mitophagy (Jin et al., 2010; Matsuda et al., 2010; Narendra et al., 2010). While PINK1‐mediated mitophagy was initially characterized in response to chemical depolarization (Jin et al., 2010; Matsuda et al., 2010; Narendra et al., 2010), PINK1 has also been shown to be activated by genetic causes of depolarization (Narendra et al., 2010; Thayer et al., 2026), oxidative stress (Wang et al., 2012), and mitochondrial proteostatic stress (Burman et al., 2017; Fiesel et al., 2017; Jin & Youle, 2013; Michaelis et al., 2022; Thayer et al., 2026).

Upon stabilization, PINK1 associates with the TOM complex and phosphorylates pre‐existing ubiquitin molecules on OMM proteins to recruit cytosolic Parkin (Callegari et al., 2025; Eldeeb et al., 2024; Lazarou et al., 2012; Okatsu et al., 2013; Okatsu et al., 2015; Raimi et al., 2024). Parkin is activated by both binding to phospho‐ubiquitin and by direct phosphorylation by PINK1 (Gladkova et al., 2018). Overall, the PINK1–Parkin pathway labels damaged mitochondria with ubiquitin chains to recruit the cellular autophagy machinery (Harper et al., 2018).

Beyond PINK1, several receptors also facilitate stress‐activated mitophagy (Lee‐Glover & Shutt, 2024; Wang et al., 2023). Some of these receptors may directly monitor protein import. For example, loss of mitochondrial membrane potential causes the matrix proteins NIPSNAP1 and NIPSNAP2 to be retained on the OMM, where they recruit the autophagic machinery (Abudu et al., 2019). Similarly, NLRX1 is normally imported into the mitochondria but under conditions of protein import stress, it remains in the cytosol and activates mitophagic signaling (Killackey et al., 2022). Notably, depletion of NIPSNAP1 and NLRX1 was demonstrated to reduce mitophagy rate in zebrafish and mice, respectively (Abudu et al., 2019; Killackey et al., 2022). Together with PINK1, these pathways monitor mitochondrial health by sensing the organelle's ability to maintain efficient protein import.

Given that both ISR and mitophagy activation can be induced by defects in mitochondrial protein import, recent studies have begun to explore crosstalk between these pathways (Chakrabarty et al., 2024; Singh et al., 2025; Yang et al., 2024). These findings reveal a complex relationship that may be context‐dependent. On one hand, HRI and phosphorylated eIF2α were shown to localize to mitochondria downstream of DELE1 activation and to promote both PINK1‐dependent and ‐independent mitophagy (Chakrabarty et al., 2024). On the other hand, ISR activation has been reported to negatively regulate mitophagy by limiting PINK1 accumulation through multiple mechanisms, including repression of its transcription and translation and enhanced mitochondrial import (Singh et al., 2025; Yang et al., 2024). Overall, these findings suggest that the interplay between mitophagy and ISR may vary depending on cell type and the nature and severity of the stress.

## 6 CONCLUDING REMARKS

Mitochondrial functions are compromised in various physiological and pathological conditions, many of which lead to inhibition of protein import. A growing body of work demonstrates the importance of surveillance mechanisms in ameliorating damage caused by unimported proteins and in maintaining mitochondrial and cellular health (Lee‐Glover & Shutt, 2024; Pfanner et al., 2025). However, many questions remain unresolved. One outstanding question is how parallel pathways interact to repair damaged mitochondria and restore protein import. For example, activation of the ISR is directly linked to defective protein import and dysfunctional mitochondria. At the same time, the ISR is a broad stress response that can be triggered by diverse insults, including ER stress, amino acid deprivation, and viral infection (Ryoo, 2024). How this general stress program tailors its output to distinct stressors remains unclear. Emerging evidence suggests that while a core set of genes is consistently regulated by the ISR across conditions, individual stressors also elicit distinct transcriptional programs (Kelley et al., 2025). The mechanisms underlying this specificity are poorly understood and could involve crosstalk between the ISR and other stress‐responsive pathways.

Another emerging topic concerns whether mitochondrial stress responses are spatially regulated within cells. The existence of distinct mitochondrial subpopulations has been described recently, raising the possibility that quality control machineries are selectively targeted to specific organelles within a cell (Ryu et al., 2024). While some factors, such as PINK1, accumulate locally to label only damaged mitochondria (Narendra et al., 2010), the mechanism for spatial regulation of other pathways is largely unexplored. This question is particularly intriguing in the context of protein import, which could enable tailoring of the mitochondrial proteome within distinct subpopulations to meet specific metabolic demands. Protein import regulators such as FNIP1 and March5 may contribute to this specificity, but whether they selectively permit the import of certain proteins while excluding others remains unclear (McMinimy et al., 2024; Phu et al., 2020).

Overall, failure to activate or properly regulate mitochondrial quality control pathways can have severe consequences and contribute to the progression of human diseases, including neurodegenerative and metabolic disorders. As such, continued efforts to understand these pathways in human cells are both timely and essential.

## AUTHOR CONTRIBUTIONS

Hilla Weidberg, Madeleine Goldstein, and Laurie Lee‐Glover wrote the manuscript.

## Data Availability

Data sharing not applicable to this article as no datasets were generated or analysed during the current study.
